# Pegcetacoplan for Treating Paroxysmal Nocturnal Haemoglobinuria: An Evidence Review Group Perspective of a NICE Single Technology Appraisal

**DOI:** 10.1007/s41669-023-00408-z

**Published:** 2023-05-17

**Authors:** Rebecca Bresnahan, Rachel Houten, Janette Greenhalgh, Sarah Nevitt, James Mahon, Sophie Beale, Angela Boland, Devarshi Bhattacharyya, Yenal Dundar, Joanne McEntee, Shreyans Gandhi, Nigel Fleeman, Marty Chaplin

**Affiliations:** 1 Liverpool Reviews and Implementation Group, University of Liverpool, Whelan Building, The Quadrangle, Brownlow Hill, Liverpool, L69 3GB UK; 2 Coldingham Analytical Services, Berwickshire, UK; 3 Hare Research, North Yorkshire, UK; 4 North West Medicines Information Centre, Liverpool, UK; 5 Kings College Hospital NHS Foundation Trust, London, UK

## Abstract

As part of the Single Technology Appraisal (STA) process, the UK National Institute for Health and Care Excellence (NICE) invited Apellis Pharmaceuticals/Sobi to submit evidence for the clinical and cost effectiveness of pegcetacoplan versus eculizumab and pegcetacoplan versus ravulizumab for treating paroxysmal nocturnal haemoglobinuria (PNH) in adults whose anaemia is uncontrolled after treatment with a C5 inhibitor. The Liverpool Reviews and Implementation Group at the University of Liverpool was commissioned as the Evidence Review Group (ERG). The company pursued a low incremental cost-effectiveness ratio (ICER) Fast Track Appraisal (FTA). This was a form of STA processed in a shorter time frame and designed for technologies with company base-case ICER < £10,000 per quality-adjusted life-year (QALY) gained and most plausible ICER < £20,000 per QALY gained. This article summarises the ERG’s review of the company's evidence submission, and the NICE Appraisal Committee’s (AC’s) final decision. The company presented clinical evidence from the PEGASUS trial that assessed the efficacy of pegcetacoplan versus eculizumab. At Week 16, patients in the pegcetacoplan arm had statistically significantly greater change from baseline in haemoglobin levels and a higher rate of transfusion avoidance than patients in the eculizumab arm. Using the PEGASUS trial and Study 302 data (a non-inferiority trial that assessed ravulizumab versus eculizumab), the company conducted an anchored matching-adjusted indirect comparison (MAIC) to indirectly estimate the efficacy of pegcetacoplan versus ravulizumab. The company identified key differences between trial designs and populations that could not be adjusted for using anchored MAIC methods. The company and ERG agreed that the anchored MAIC results were not robust and should not inform decision making. In the absence of robust indirect estimates, the company assumed that ravulizumab had equivalent efficacy to eculizumab in the PEGASUS trial population. Results from the company base-case cost-effectiveness analysis showed that treatment with pegcetacoplan dominated eculizumab and ravulizumab. The ERG considered that the long-term effectiveness of pegcetacoplan was uncertain and ran a scenario assuming that after 1 year the efficacy of pegcetacoplan would be the same as eculizumab; treatment with pegcetacoplan continued to dominate eculizumab and ravulizumab. The AC noted that treatment with pegcetacoplan had lower total costs than treatment with eculizumab or ravulizumab because it is self-administered and reduces the need for blood transfusions. If the assumption that ravulizumab has equivalent efficacy to eculizumab does not hold, then this will affect the estimate of the cost effectiveness of pegcetacoplan versus ravulizumab; however, the AC was satisfied that the assumption was reasonable. The AC recommended pegcetacoplan as an option for the treatment of PNH in adults who have uncontrolled anaemia despite treatment with a stable dose of a C5 inhibitor for ≥ 3 months. Pegcetacoplan was the first technology recommended by NICE via the low ICER FTA process.

## Key Points for Decision Makers

**Table:** 

Data for pegcetacoplan versus eculizumab were only available from the PEGASUS trial; the trial included a small number of patients (N=80) and maximum follow-up was 48 weeks.
The company carried out an anchored matching-adjusted indirect comparison (MAIC) to provide indirect evidence for pegcetacoplan versus ravulizumab; however, the Evidence Review Group (ERG) and company agreed that MAIC results should not be used to inform decision making and so the company assumed that ravulizumab had equivalent efficacy to eculizumab.
The ERG and National Institute for Health and Care Excellence (NICE) Appraisal Committee (AC) were satisfied that if the efficacy of ravulizumab was equal to that of eculizumab, the most plausible low incremental cost-effectiveness ratios (ICERs) per quality-adjusted life year (QALY) gained for pegcetacoplan versus eculizumab and pegcetacoplan versus ravulizumab were <£20,000.

## Introduction

The National Institute for Health and Care Excellence (NICE) is an independent organization that provides national guidance to the National Health Service (NHS) in England and Wales on a range of clinical and public health issues, including the appraisal of new health technologies. The NICE Single Technology Appraisal (STA) process is designed for the appraisal of a single health technology for a single indication, where most of the relevant evidence lies with one manufacturer or sponsor. Typically, the process is used for new pharmaceutical products close to launch.

Within the STA process, the company provides a written submission (alongside a decision analytic model) that summarises an estimate of the clinical and cost effectiveness of the technology (the company submission). An external independent organisation (typically, an academic group), previously known as an Evidence Review Group (ERG) and now known as the External Assessment Group (EAG), provides a critique of the company’s submission (the ERG report). Consultees, clinical specialists and patient representatives also provide additional information during the appraisal process.

Using a specification developed by NICE (the final scope), the NICE Appraisal Committee (AC) considers the company’s submission, the ERG report, and testimonies from experts and stakeholders to determine whether the technology represents a clinically effective and cost-effective use of NHS resources. All stakeholders and the public have an opportunity to comment on the preliminary guidance issued by NICE (the Appraisal Consultation Document [ACD]), after which the NICE AC meets again to produce the final guidance (Final Appraisal Determination [FAD]). The final guidance constitutes a legal obligation for NHS providers in England and Wales to provide a technology that is recommended within its licensed indication.

From April 2017 to February 2022, NICE methods specified that eligible technologies could proceed via the Fast Track Appraisal (FTA) process. This was a form of the STA process, undertaken over a shorter time frame to allow faster patient access to the most cost-effective treatments [1]. Technologies were eligible for the FTA process if they were considered to offer exceptional value for money (low incremental cost-effectiveness ratio [ICER] FTA) or were likely to provide similar or greater health benefits at similar or lower costs than technologies already recommended via the STA process for the same indication (cost comparison FTA). A technology was appraised via the low ICER FTA process if the company base case was < £10,000 per quality-adjusted life-year (QALY) gained and if the most plausible ICER per QALY gained was < £20,000 and highly unlikely to be > £30,000.

Pegcetacoplan was the first technology to be recommended by NICE via the low ICER FTA process. This article includes a summary of the ERG report and the AC’s final decision for the FTA of pegcetacoplan for treating paroxysmal nocturnal haemoglobinuria (PNH) in adults whose anaemia is uncontrolled after treatment with a C5 complement inhibitor. The Liverpool Reviews and Implementation Group at the University of Liverpool was commissioned to act as the ERG for this appraisal. Full details of all the relevant appraisal documents (including the appraisal scope, ERG report, company and consultee submissions, final appraisal document and comments on each of these) can be found on the NICE website [2].

## Decision Problem

PNH is a rare, acquired, life-threatening chronic blood condition [3]. It is caused by a loss of function mutation in bone marrow stem cells that leads to production of abnormal red blood cells [3]. The abnormal red blood cells lack CD55 and CD59, two surface proteins that regulate the activity of the complement system (part of the immune system that consists of more than 30 proteins) [4]. As a consequence, red blood cells become vulnerable to attack from the complement system, including the complement components C3 and C5 [4]. This leads to destruction of red blood cells (haemolysis) and formation of blood clots (thrombosis) [3]. Haemolysis can occur within the vasculature (intravascular haemolysis [IVH]) or in the liver, spleen, bone marrow or lymph nodes (extravascular haemolysis [EVH]) [3].

PNH can be acquired at any age but is most frequently diagnosed in adults aged 30–40 years [5]. It is estimated that the global incidence of PNH is 1–2 cases per million population per year [6, 7] and that the global prevalence is between 12 per million population [8] and 16 per million population [7]. Approximately 15% of patients experience spontaneous remission, most commonly 10–20 years after diagnosis [9].

**Fig. 1:**
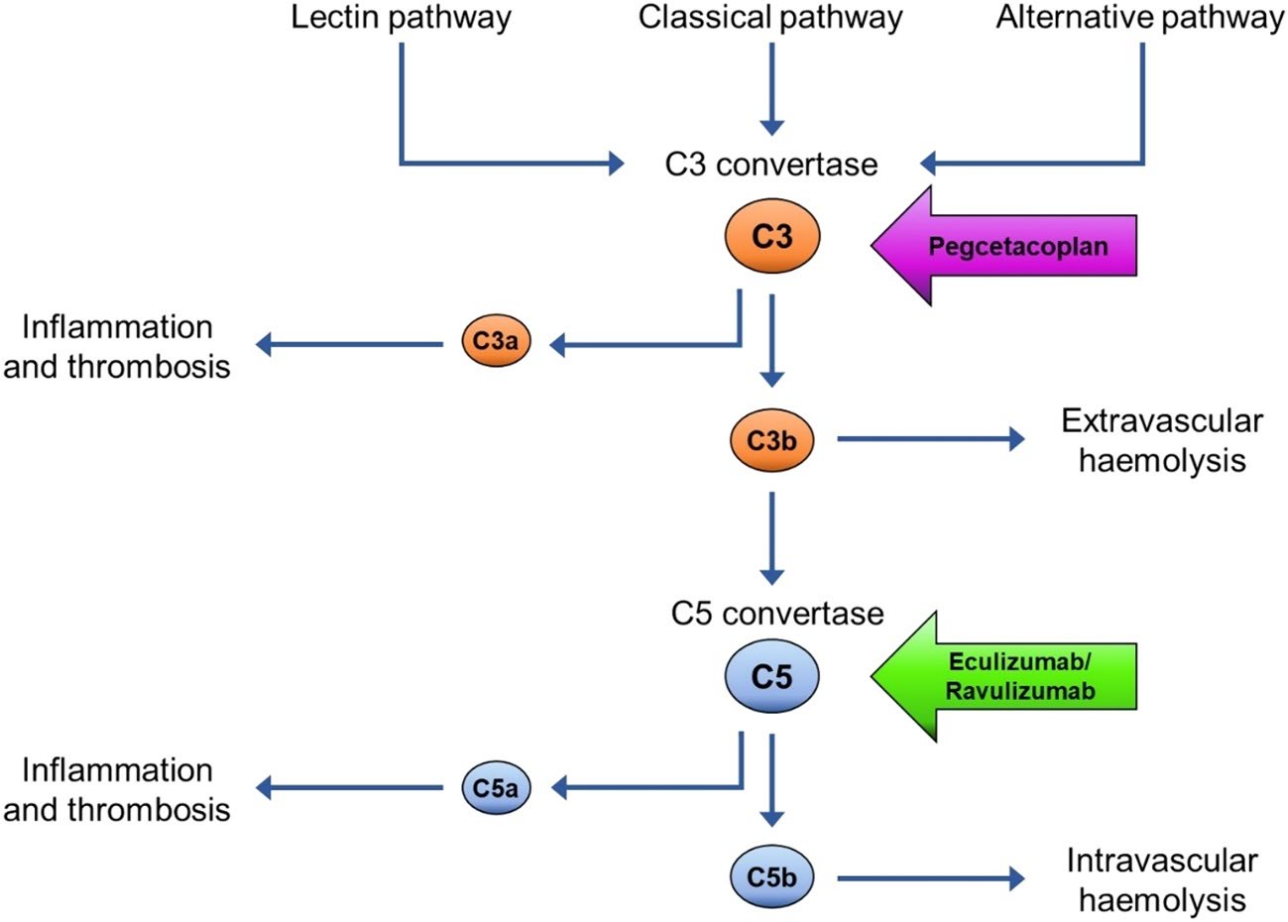
Mechanism of action for C3 inhibitors and C5 inhibitors. The complement system can be activated via the lectin, classical or alternative pathway. All three pathways result in the formation of C3 convertase. Active C3 convertase cleaves complement protein C3 into C3a and C3b. Once active, C3a leads to inflammation and thrombosis and C3b leads to extravascular haemolysis (EVH). C3b is also a component of C5 convertase which cleaves complement protein C5 into C5a and C5b. Similar to C3a, C5a leads to inflammation and thrombosis. C5b leads to intravascular haemolysis (IVH). Pegcetacoplan is a C3 inhibitor that binds to C3 to prevent its cleavage by C3 convertase. Pegcetacoplan targets early in the complement cascade and therefore prevents IVH and EVH. C5 inhibitors (eculizumab and ravulizumab) bind to C5 to prevent cleavage by C5 convertase. C5 inhibitors act later in the complement cascade and therefore only prevent IVH [12]

Treatment with a C5 inhibitor (i.e., eculizumab or ravulizumab) prevents IVH but does not prevent EVH [10]. Pegcetacoplan is an inhibitor of complement proteins C3 and C3b which are activated earlier in the complement cascade than C5. Pegcetacoplan is therefore able to prevent EVH and IVH (Fig. 1). Pegcetacoplan is a self-administered, twice weekly (1080 mg subcutaneous [SC]) infusion and is licensed in Europe for the treatment of adult patients with PNH whose anaemia is uncontrolled after treatment with a C5 inhibitor for ≥ 3 months [11].

NICE developed a scope [13] for the assessment of pegcetacoplan, in which it was specified that the clinical and cost effectiveness of pegcetacoplan should be established within its licensed indication, relative to established clinical practice, that is, treatment with eculizumab or ravulizumab. Nine measures of clinical effectiveness were considered relevant for this appraisal: overall survival (OS), IVH, EVH, breakthrough haemolysis (BTH), transfusion avoidance, haemoglobin (Hb) outcomes, thrombotic events, adverse events (AEs) of treatment and health-related quality of life (HRQoL). The time horizon of analysis was specified as the remaining lifetime of patients.

## The Independent ERG Report

In May 2021, the company (Apellis Pharmaceuticals/Sobi) provided a submission to NICE describing the use of pegcetacoplan (within the context of its licensed indication) in patients with PNH who had uncontrolled anaemia after treatment with a C5 inhibitor for a period of ≥ 3 months. The ERG produced a report that comprised a critical review of the clinical effectiveness and cost-effectiveness evidence included in the company’s submissions to NICE, including the company’s response to the ERG’s request for clarification on a number of issues.

The ERG review

- assessed whether the company’s submission conformed to NICE methodological guidelines;
- assessed whether the company’s interpretation and analysis of the evidence were appropriate;
- highlighted the presence of other sources of evidence or provided alternative interpretations of the evidence that could help inform the development of NICE guidance.

In addition to providing this detailed critique, the ERG modified a number of key assumptions and parameters within the company’s economic model to examine the impact of making these changes. Sections 3.1 to 3.5 include summaries of the evidence submitted by the company and the ERG’s review of that evidence.

### Clinical Evidence

#### Direct Evidence

**Fig. 2:**
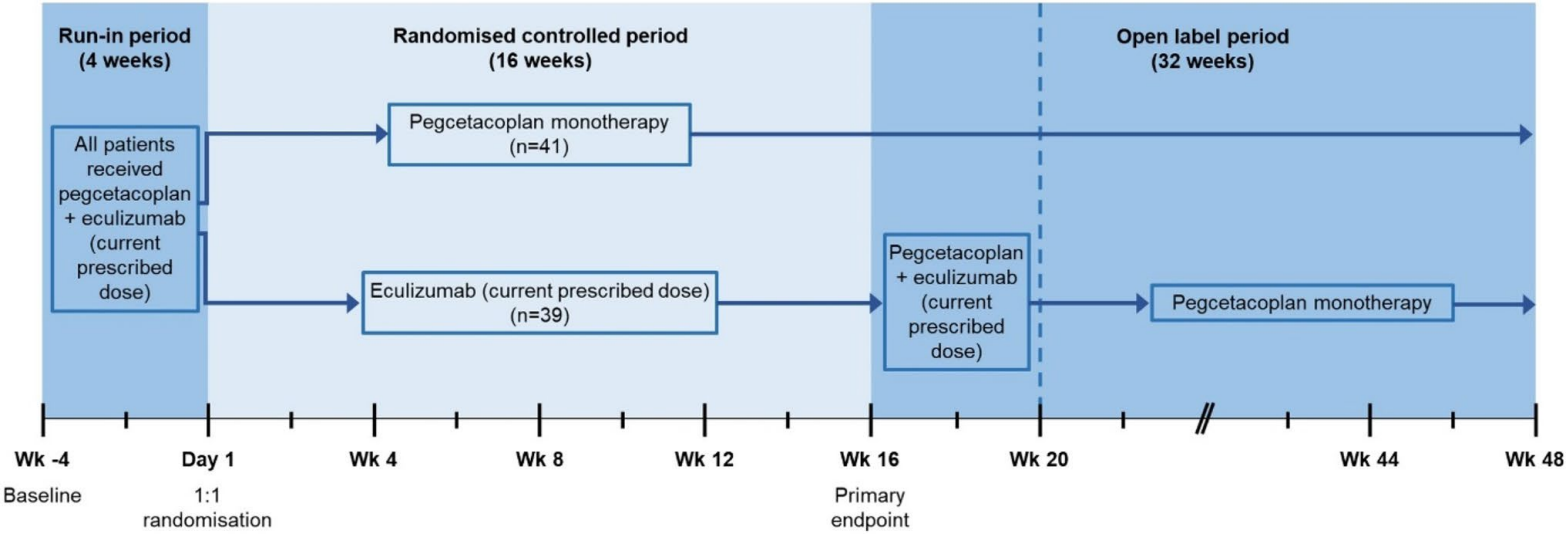
The PEGASUS trial design. In the PEGASUS trial, Day − 28 (Week − 4) represented baseline. During the run-in period (Week − 4 to Day 0), all patients received pegcetacoplan plus eculizumab at their current prescribed dose. On completion of the run-in period, patients entered the randomised controlled period (RCP) and were randomised to receive pegcetacoplan monotherapy (*n* = 41) or to stop pegcetacoplan and only receive their current prescribed dose of eculizumab (*n* = 39) for 16 weeks (Day 1 to Week 16). Patients who completed the RCP were invited to enter the 32-week open-label period (OLP). Patients randomised to pegcetacoplan monotherapy continued to receive pegcetacoplan monotherapy (Week 16 to Week 48). Patients randomised to eculizumab were required to repeat the 4-week run-in period (Week 16 to Week 20) before switching to pegcetacoplan monotherapy (Week 20 to Week 48)

The company conducted a literature search that identified a single randomised controlled trial (RCT), the PEGASUS trial [14]. The PEGASUS trial [14] was a 48-week, multicentre, international, randomised, open-label, active-comparator, phase III RCT designed to assess the clinical effectiveness of pegcetacoplan at the licensed dose (1080 mg self-administered subcutaneously [SC] twice weekly or every 3 days) compared with patients’ current prescribed eculizumab dosage (intravenous [IV] infusion; range: 900–1500 mg every 2 weeks) for patients with PNH whose anaemia was not controlled after treatment with a C5 inhibitor. The PEGASUS trial [14] included 80 patients (≥ 18 years old) with PNH who continued to have Hb levels < 10.5 g/dL despite treatment with eculizumab. Eligible patients had received a stable eculizumab dose for ≥ 3 months prior to screening. The trial consisted of three phases: a 4-week run-in period, a 16-week randomised controlled period (RCP) and a 32-week open-label period (OLP). The trial design is summarised in Fig. 2. The PEGASUS trial [14] did not compare pegcetacoplan with ravulizumab, the other comparator specified in the NICE scope [13].

#### Direct Evidence for Pegcetacoplan Versus Eculizumab

The company presented evidence from the PEGASUS trial RCP [14] (24th December 2019 database lock). At the time of the database lock, the PEGASUS trial [14] included 41 patients randomised to the pegcetacoplan arm and 39 patients randomised to the eculizumab arm. All patients had completed their Week 16 visit or had discontinued prior to Week 16.

The direct evidence from the PEGASUS trial [14] is presented in Tables 1 and 2. Data were analysed for an intention-to-treat population at Week 16 of the RCP using the mixed model repeated measures (MMRM) approach and were censored for transfusion (i.e., subsequent outcome measurements were set to missing following a transfusion). The company clarification response included the observed values and change from baseline (CFB) at Week 16 results without censoring for transfusion for the following outcomes: Hb level, absolute reticulocyte count (ARC), ARC normalisation, lactate dehydrogenase (LDH) level and indirect bilirubin level. The ERG considered that the uncensored values were consistent with the censored values. The company also provided the observed values and CFB without censoring for transfusion for all reported outcomes for the OLP (Week 17 to Week 48). Data for the OLP were academic in confidence at the time of publishing the ERG report but have since been made publicly available [15–17]. Patient OS was not assessed as part of the PEGASUS trial [14]. Clinical advice to the ERG was that mortality hazards for treated patients are the same as those for the general population.

**Table 1:** Summary of PEGASUS trial change from baseline at Week 16 results: intention-to-treat population (mixed model repeated measures model, censored for transfusion). Source: Company submission [18] and clarification response [19]

	LS mean (SE)		LS mean difference (95% CIs)	*p* value
	Pegcetacoplan (*n* = 41)	Eculizumab (*n* = 39)		
CFB in Hb level, g/dL	2.37 (0.36)	− 1.47 (0.67)	3.84 (2.33 to 5.34)	< 0.0001
CFB in ARC, 10^9^ cells/L	− 135.82 (6.54)	27.79 (11.86)	− 163.61 (− 189.91 to −137.30)	< 0.0001
CFB in LDH level, U/L	− 14.76 (42.71)	− 10.12 (71.03)	−4.63 (− 181.30 to 172.04)	0.9557
CFB in indirect bilirubin level, µmol/L	− 17.78 (2.73)	4.15 (4.48)	− 21.93 (− 32.49 to − 11.36)	0.0002

*ARC* absolute reticulocyte count normalisation, *CFB* change from baseline, *CI* confidence interval, *LDH* lactate dehydrogenase, *LS* least squares, *SE* standard error

**Table 2:** Summary of PEGASUS trial outcomes at Week 16 results: intention-to-treat population. Source: Company submission [18]

	Pegcetacoplan (*n* = 41)	Eculizumab (*n* = 39)	Risk difference (95% CI)	*p* value
Transfusion avoidance, *n* (%)^a^	35 (85.4)	6 (15.4)	0.63 (0.48–0.77)	< 0.0001
Hb response in absence of transfusion, *n* (%)^b^	31 (75.6)	0 (0.0)	0.68 (0.55–0.80)	NR
Hb normalisation in absence of transfusion, *n* (%)^c^	14 (34.1)	0 (0.0)	0.30 (0.15–0.46)	NR
ARC normalisation in absence of transfusion, *n* (%)^d^	32 (78.0)	1 (2.6)	0.66 (0.53–0.80)	NR
LDH normalisation in absence of transfusion, *n* (%)^e^	29 (70.7)	6 (15.4)	NR	NR

*ARC* absolute reticulocyte count normalisation, *CI* confidence interval, *Hb* haemoglobin, *LDH* lactate dehydrogenase, *LLN* lower limit of normal, *NR* not reported, *ULN* upper limit of normal

^a^Number of patients who did not receive a transfusion

^b^Defined as an increase of ≥ 1 g/dL from baseline Hb level at Week 16 without transfusion

^c^Defined as Hb level ≥ the gender-specific LLN range (female LLN: 12 g/dL; male LLN: 13.6 g/dL) at Week 16 without transfusion

^d^Defined as ARC < 120 × 10^9^ cells/L (ULN) at Week 16 without transfusion

^e^Defined as LDH level < 226 U/L (ULN) at Week 16 without transfusion

##### Efficacy Outcomes

The primary objective of the PEGASUS trial [14] was to assess CFB in Hb levels at Week 16 of the RCP. CFB in Hb levels was statistically significantly greater in the pegcetacoplan arm at Week 16 compared with the eculizumab arm (least squares [LS] mean difference: 3.84, 95% confidence interval [CI]: 2.33–5.34, *p* < 0.0001; Table 1). The observed Hb levels were higher in the pegcetacoplan arm compared with the eculizumab arm at all time points when data were censored for transfusion.

Transfusion avoidance during the RCP was statistically significantly higher in the pegcetacoplan arm compared with the eculizumab arm (risk difference [RD]: 0.63, 95% CI 0.48 to 0.77, *p* < 0.0001; Table 2). Non-inferiority was demonstrated (as the lower bound of the 95% CI exceeded the pre-defined non-inferiority margin of − 20%) for pegcetacoplan versus eculizumab. At Week 48, 30/41 patients (73.2%) in the pegcetacoplan arm had not required a transfusion during the RCP or OLP.

ARC and indirect bilirubin level are biomarkers of EVH. Indirect bilirubin level is also a biomarker of IVH, but to a lesser extent. Reduced ARC and indirect bilirubin level indicate reduced EVH. At Week 16, ARC (LS mean difference: − 163.61 × 10^9^ cells/L, 95% CI − 189.91 to − 137.30, *p* < 0.0001) and indirect bilirubin level (LS mean difference: − 21.93 µmol/L, 95% CI − 32.49 to − 11.36, *p* = 0.0002) were statistically significantly reduced compared with baseline in the pegcetacoplan arm versus the eculizumab arm (Table 1). Non-inferiority was demonstrated for ARC (as the upper bound of the 95% CI was less than the pre-defined non-inferiority margin of 10 × 10^9^ cells/L) for pegcetacoplan versus eculizumab but was not reported for indirect bilirubin level.

LDH level is a biomarker of IVH. Reduced LDH level indicates reduced IVH. CFB in LDH level at Week 16 was similar in the pegcetacoplan (− 14.76 U/L) and eculizumab (− 10.12 U/L) arms (LS mean difference: − 4.63 U/L, 95% CI − 181.30 to 172.04, *p* = 0.9557; Table 1). Pegcetacoplan did not demonstrate non-inferiority versus eculizumab (as the upper bound of the 95% CI was not less than the pre-defined non-inferiority margin of 20 U/L). Clinical advice to the ERG was that LDH levels in the pegcetacoplan and eculizumab arms were well controlled at baseline (< 1.5 × upper limit of normal [ULN]; < 339 U/L) and remained well controlled at Week 16.

At Week 16, more patients in the pegcetacoplan arm achieved a Hb response, Hb normalisation, ARC normalisation and LDH normalisation than in the eculizumab arm (Table 2). Patients in the pegcetacoplan arm maintained observed Hb levels, ARC and indirect bilirubin levels from Week 16 of the RCP to Week 48 of the OLP. The observed LDH levels fluctuated from Week 16 to Week 48 but the mean observed LDH level remained below 1.5 × ULN.

##### Health-Related Quality of Life

Health-related quality of life (HRQoL) data were collected during the PEGASUS trial [14] using three instruments: the European Organisation for Research and Treatment of Cancer Quality of Life Questionnaire-Core 30 (EORTC QLQ-C30) (v0), the Functional Assessment of Chronic Illness Therapy (FACIT)-Fatigue scale (v4) and the Linear Analog Scale Assessment (LASA).

For the pegcetacoplan arm, EORTC QLQ-C30 global health status (GHS)/quality of life (QoL) score had increased by 15.44 (standard deviation [SD]: 3.05) at Week 16 (a 10-point increase is generally considered to be a clinically meaningful improvement) and patients improved on all functional scales. In contrast, mean GHS/QoL score at Week 16 had decreased in the eculizumab arm (− 3.83, SD: 3.13) compared with baseline.

From Week 2 onwards, the observed mean FACIT-Fatigue score (censored for transfusion) for the pegcetacoplan arm was comparable to scores derived from the general population (43.38 and 43.60, respectively). At Week 16, patients in the pegcetacoplan arm had greater mean FACIT-fatigue (LS mean numerical difference: 11.87, 95% CI 5.49–18.25) and LASA scores (LS mean numerical difference: 59.10, 95% CI 16.88–101.32) compared with the eculizumab arm. An increase of 3 points in FACIT-fatigue score [16] and an increase of 30–60 points in LASA score [20] are considered clinically meaningful improvements.

Patients in the pegcetacoplan arm maintained improved EORTC QLQ-C30 GHS/QoL, FACIT-Fatigue and LASA scores from Week 16 of the RCP to Week 48 of the OLP.

##### Safety and Tolerability

During the run-in period, where both treatment arms received pegcetacoplan, there were no treatment-emergent adverse events (TEAEs) reported leading to study or treatment discontinuation, or death. During the RCP, similar proportions of patients in the pegcetacoplan and eculizumab arms experienced at least one TEAE (87.8% and 87.2%, respectively). During the RCP, 7/41 patients (17.1%) in the pegcetacoplan arm and 6/39 patients (15.4%) in the eculizumab arm experienced serious TEAEs. Of these, one patient in each arm experienced a treatment-related AE. Injection-site reaction was the most common TEAE for patients in the pegcetacoplan arm (15/41, 36.6%; Table 3). During the RCP, 3/41 patients (7.3%) in the pegcetacoplan discontinued pegcetacoplan due to BTH. No thromboembolic events or deaths were reported.

**Table 3:** Summary of treatment-emergent adverse events during the randomised controlled period: safety population

	Pegcetacoplan (*n* = 41)	Eculizumab (*n* = 39)
Any TEAEs, *n* (%)	36 (87.8)	34 (87.2)
Mild	19 (46.3)	14 (35.9)
Moderate	9 (22.0)	15 (38.5)
Severe	8 (19.5)	5 (12.8)
Serious TEAEs, *n* (%)	7 (17.1)	6 (15.4)
Injection-site reaction, *n* (%)	15 (36.6)	1 (2.6)
TEAEs leading to study drug discontinuation, *n* (%)	3 (7.3)^a^	0
TEAEs leading to death, *n* (%)	0	0

*TEAE* treatment-emergent adverse event

^a^3/41 patients in the pegcetacoplan arm discontinued pegcetacoplan due to breakthrough haemolysis

#### Indirect Evidence for the Efficacy of Pegcetacoplan Versus Ravulizumab

In the absence of head-to-head data comparing the efficacy and safety of pegcetacoplan with ravulizumab, the company conducted an anchored MAIC using PEGASUS trial [14] and Study 302 [21] data.

Study 302 [21] was a randomised, open-label, multicentre, phase III non-inferiority study which compared the clinical efficacy of ravulizumab versus eculizumab for adult patients with PNH who had previously been treated with eculizumab. The company adjusted individual patient data (IPD) from the PEGASUS trial [14] to match the aggregate baseline characteristics of Study 302 [21]. The indirect comparison of pegcetacoplan and ravulizumab was anchored by the common eculizumab control arm.

The company considered that the definitions of the following outcomes measured in the PEGASUS trial [14] and Study 302 [21] were similar enough to be included in the anchored MAIC: transfusion avoidance, transfusion requirements (total number of units of packed red blood cells transferred), Hb stabilisation (avoidance of ≥2 g/dL decrease in Hb levels) and LDH normalisation in the absence of transfusion, FACIT-fatigue score and EORTC QLQ-C30 scores (general health status, physical functioning and fatigue symptoms).

The anchored MAIC results showed statistically significant advantages for pegcetacoplan over ravulizumab for all outcomes considered. However, the company identified key differences between the trial designs and populations that could not be adjusted for using anchored MAIC methods (or any other adjusted indirect comparison method) and did not use the anchored MAIC results in their economic model. Differences included treatment phases, lengths of treatment periods (16 weeks in the PEGASUS trial [14] versus 26 weeks in Study 302 [21]), administration routes and schedules for pegcetacoplan and ravulizumab, eculizumab dose and eligibility criteria. Study 302 [21] included patients who were ‘clinically stable’ on eculizumab and had received eculizumab at the licensed dose (900 mg every 2 weeks) for ≥ 6 months prior to screening. In Study 302 [21], patients were eligible for inclusion regardless of Hb levels. CFB in Hb level, the primary outcome of the PEGASUS trial [14], was not measured in Study 302 [21]. The company and the ERG agreed with the authors of the NICE Decision Support Unit (DSU) Technical Support Document (TSD) 18 report [22] that exclusion of important effect modifiers from the matching process (in this case, Hb levels and history of transfusions) meant that anchored MAIC results were biased. The ERG agreed with the company conclusion that anchored MAIC results were not robust and should not be used to inform decision making.

#### ERG Critique of Clinical Evidence

The ERG was satisfied that the methods used by the company to conduct a systematic review of the clinical effectiveness evidence were appropriate. The ERG did not identify any additional studies that should have been included in the company’s review. The ERG considered that the PEGASUS trial [14] was well designed and well conducted and that the company’s statistical approach was pre-specified and appropriate.

In line with the NICE scope [13], the company presented clinical effectiveness evidence for patients with PNH who had uncontrolled anaemia after treatment with a C5 inhibitor for ≥ 3 months. The term ‘uncontrolled’ was not defined in the NICE scope [13]; however, at baseline, patients enrolled in the PEGASUS trial had Hb levels < 10.5 g/dL and the company appeared to assume that these patients could be considered to have anaemia that was uncontrolled. Clinical advice to the ERG was that in NHS clinical practice, some PNH patients with Hb levels ≥ 10.5 g/dL may also be considered to have uncontrolled anaemia.

The company and the ERG agreed that the baseline characteristics of patients in the PEGASUS trial [14] were well balanced across the treatment arms. Clinical advice to the ERG was that approximately 20% of patients in NHS clinical practice have a suboptimal response (i.e., no change to transfusion requirements) to eculizumab and that patients in the PEGASUS trial [14] are representative of this population. Clinical advice to the ERG was that approximately 50% of patients with PNH have some underlying bone marrow failure (e.g., aplastic anaemia). In these patients, anaemia is not due to uncontrolled complement activity and is unlikely to respond to higher doses of C5 or C3 inhibitors.

The ERG’s key area of concern was the absence of direct evidence (and only biased indirect evidence) to demonstrate the effectiveness of pegcetacoplan versus ravulizumab. The NICE recommendation for ravulizumab [23] was based on results from Study 302 [21] which showed that ravulizumab was non-inferior to eculizumab, with point estimates favouring ravulizumab for all primary and key secondary endpoints. Further expert advice to the ERG was that most patients who were treated with eculizumab prior to NICE recommending ravulizumab were likely to switch to treatment with ravulizumab due to reduced treatment burden and improved patient convenience associated with ravulizumab (infusions every 8 weeks for ravulizumab versus every 2 weeks for eculizumab).

### Cost-Effectiveness Evidence

#### Company’s Economic Evidence

The company model was constructed in MS Excel and was used to compare the cost effectiveness of pegcetacoplan versus eculizumab and pegcetacoplan versus ravulizumab in patients with PNH who had baseline Hb levels < 10.5 g/dL despite treatment with a stable dose of a C5 inhibitor for ≥ 3 months. The company stated that, in line with the NICE Reference Case [24], costs were calculated from the perspective of the NHS and personal social services (PSS). The model cycle length was 4 weeks, the model time horizon was 51 years (to represent patients’ remaining lifetime), and costs and outcomes were discounted at 3.5% per annum.

The model was a cohort-based Markov model comprising four mutually exclusive health states: No Transfusion (in previous 4 weeks) and Hb levels < 10.5 g/dL, No Transfusion (in previous 4 weeks) and Hb levels ≥ 10.5 g/dL, Transfusion Required (in previous 4 weeks) and Death. The Hb level cut-off (10.5 g/dL) used in the model was consistent with the PEGASUS trial [14] inclusion criterion.

Patient-level data from the PEGASUS trial [14] were used by the company to estimate base-case transition probabilities for patients receiving pegcetacoplan and eculizumab (pegcetacoplan: baseline to Week 48; eculizumab: baseline to Week 16). Ravulizumab was assumed to have equivalent efficacy to eculizumab. A multinomial logistic regression model with the current health state as the outcome variable and age, visits, treatment and health as covariates was used to calculate transition probabilities. Based on PEGASUS trial [14] data, some patients were modelled to discontinue treatment with pegcetacoplan and subsequently restart treatment with eculizumab at Week 16.

The company defined BTH as one or more new or worsening symptom(s) or sign(s) of IVH or EVH. Expert advice to the company was that the decrease in Hb levels and blood transfusions resulting from extravascular breakthrough haemolysis (EVBTH) were captured in the model health states and, therefore, it was not necessary to explicitly model EVBTH.

At the time of the PEGASUS trial [14], there was no established approach to treating intravascular breakthrough haemolysis (IVBTH) for patients treated with pegcetacoplan; however, expert advice to the company was that patients treated with pegcetacoplan who experienced IVBTH would be prescribed a one-off dose of eculizumab (900 mg). The cost of one-off treatment with eculizumab was included in the company model. IVBTH was not modelled for patients receiving eculizumab or ravulizumab; the company assumed that for patients treated with these drugs, IVBTH would be managed using dose adjustments which were already captured within drug cost calculations.

In the company model, a proportion of transfusion dependent patients with EVH who were treated with eculizumab or ravulizumab were on life-long chelation therapy for iron overload. Patients treated with pegcetacoplan were assumed not to need chelation therapy as their Hb levels could be managed by phlebotomy.

In line with the NICE Reference Case [24], the company mapped PEGASUS trial [14] EORTC QLQ-C30 data to EuroQol 5-Dimensions 3-level (EQ-5D-3L) values using the Longworth et al. 2014 [25] mapping algorithm, and then made age adjustments using the Ara and Brazier 2011 [26] algorithm. The model also included a disutility to account for the effect of chelation therapy (− 0.03) and a disutility to model the effect of frequent regular eculizumab infusion (− 0.025) [27]. AE costs were not included and it was assumed that mortality was not affected by treatment.

In the company base case, treatment with pegcetacoplan dominated eculizumab and ravulizumab and the ICERs per QALY gained were below £20,000. The company therefore considered pegcetacoplan a candidate for the NICE low ICER FTA process.

#### ERG Critique of Company’s Cost-Effectiveness Evidence

In the company model, the proportion of patients who receive chelation therapy was based on the number of patients treated with desferrioxamine mesilate or deferasirox during the PEGASUS trial [14] run-in period, a period when all patients were receiving pegcetacoplan and eculizumab. Data presented in the PEGASUS trial clinical study report (CSR) [28] for the proportion of patients receiving chelation therapy differed to the values used in the company model. The ERG amended the company model inputs to reflect the PEGASUS trial CSR [28] data.

The ERG considered that the proportion of patients receiving chelation therapy during the PEGASUS trial [14] run-in period was a poor proxy for the proportion of patients who would require chelation therapy over the whole model time horizon. Studies suggest that chronic blood transfusion therapy leads to secondary iron overload and that, generally, chelation therapy with deferoxamine is started after 2–3 years of transfusions (or when a patient’s blood ferritin level exceeds 1000 ng/mL) [29]. Thus, the company assumption of limiting the proportion of patients requiring chelation therapy to the proportion who were receiving it during the run-in period may have led to an underestimation of the costs, and overestimation of the utilities, associated with treatment with eculizumab and ravulizumab. If the proportion of patients requiring chelation therapy has been underestimated, the company base-case estimates of the cost effectiveness of pegcetacoplan versus eculizumab and versus ravulizumab would be underestimates.

The company and the ERG considered that the impact of AEs on utilities would have been captured by the EORTC-QLQ-30 data (which were mapped to EQ-5D scores to generate health state utility values) and, therefore, it was not necessary to include AE-related disutilities in the model.

The ERG considered that it was not possible to be certain that, in the PEGASUS trial [14] population, the efficacy of ravulizumab would be the same as the efficacy of eculizumab. If this assumption of equivalence is not valid, the company may have over- or under-estimated the cost effectiveness of pegcetacoplan versus ravulizumab. The ERG was unable to test the consequences of varying this assumption in the company model.

The PEGASUS trial [14] was the only source of data to demonstrate the effects (in terms of efficacy or AEs) of treatment with pegcetacoplan (48 weeks) or treatment with eculizumab (16 weeks). The ERG was concerned that short-term data from a small population (*N* = 80) were used to generate transition probabilities that controlled movement between model health states over the 51-year model time horizon. The ERG explored the impact of assuming that after 1 year the efficacy of pegcetacoplan became equal to the efficacy of eculizumab. Results from this scenario analysis showed that treatment with pegcetacoplan continued to dominate eculizumab and ravulizumab.

The ERG also explored the effect of assuming a change to the proportion of patients who discontinue treatment with pegcetacoplan during Year 1. The implementation of this change had no effect on cost-effectiveness conclusions; treatment with pegcetacoplan remained dominant compared with eculizumab and ravulizumab.

Clinical advice to the ERG was that, over time, ravulizumab was likely to become the preferred C5 inhibitor for first-line treatment in most patients with PNH. It is, therefore, likely that patients who have BTH and discontinue pegcetacoplan would return to their original ravulizumab treatment rather than switch to treatment with eculizumab, as implied in the company model. The ERG did not explore the impact of this change on cost-effectiveness results but highlighted that, if ravulizumab costs more (or less) than eculizumab, the use of ravulizumab on discontinuation of pegcetacoplan would increase (or decrease) the total costs associated with BTH treatment and the consequence of this would be to increase (or decrease) the base-case ICER per QALY gained for the comparison of pegcetacoplan versus ravulizumab.

### End-of-Life Guidance Criteria

The company and the ERG agreed that pegcetacoplan did not meet End-of-Life criteria.

### Conclusions of the ERG Report

The key area of concern was the absence of direct evidence (and only biased indirect evidence) to demonstrate the effectiveness of pegcetacoplan versus ravulizumab in the PEGASUS trial [14] population. The NICE recommendation for ravulizumab [23] was based on results from Study 302 [19] (which showed that ravulizumab was non-inferior to eculizumab, with point estimates favouring ravulizumab for all primary and key secondary endpoints). However, Study 302 [19] enrolled a population that was broader than the PEGASUS trial [14] population. In addition, there were key differences between the Study 302 [19] and PEGASUS trial [14] designs.

It was unclear whether the Hb cut-off level of < 10.5 g/dL (a PEGASUS trial [14] entry criterion) was an appropriate cut-off level to determine whether PNH patients treated in NHS clinical practice have uncontrolled anaemia.

The ERG was satisfied that if the efficacy of ravulizumab was equal to the efficacy of eculizumab for patients with PNH who have baseline Hb levels < 10.5 g/dL despite treatment with a stable dose of a C5 inhibitor for ≥ 3 months, the most plausible ICERs per QALY gained for the comparisons of pegcetacoplan versus eculizumab and pegcetacoplan versus ravulizumab were < £20,000 and therefore met the NICE low ICER FTA criteria. The ERG considered there were no other critical issues relating to the economic model submitted by the company.

### Key Methodological Issues

The key methodological issue was the indirect clinical effectiveness evidence for the comparison of pegcetacoplan versus ravulizumab. The company carried out an anchored MAIC to estimate the clinical effectiveness of pegcetacoplan versus ravulizumab. The ERG agreed with the company that estimates from the anchored MAIC may have been “subject to bias” and were not suitable to inform decision making due to differences between the two included trials (the PEGASUS trial [14] and Study 302 [21]) in key effect modifiers that could not be accounted for in the matching process. To generate cost-effectiveness estimates for the comparison of the effectiveness of pegcetacoplan versus ravulizumab, an assumption was made that the effectiveness of ravulizumab was equal to that of eculizumab.

The long-term effectiveness of pegcetacoplan is uncertain. The impact of this on cost-effectiveness estimates was explored by using sensitivity analyses conducted by both the company and the ERG.

## Nice Guidance

In March 2022, NICE recommended pegcetacoplan for the treatment of PNH in adults who have uncontrolled anaemia despite treatment with a stable dose of a C5 inhibitor for ≥ 3 months [30]. This recommendation was conditional on the company providing pegcetacoplan to the NHS according to the commercial arrangement.

### New Treatment Option

The AC considered that as pegcetacoplan works by inhibiting the complement protein C3 rather than C5, it will likely target both IVH and EVH. The NICE AC recognised that pegcetacoplan could therefore offer benefit for people who continue to have uncontrolled anaemia while having C5 inhibitors that only target IVH. The NICE AC concluded that people with PNH would welcome pegcetacoplan as a new treatment option.

### Treatment Pathway

The company’s proposed positioning of pegcetacoplan meant that it would only be offered to people if their condition was not well enough controlled after treatment with eculizumab or ravulizumab.

### Clinical Evidence

The NICE AC considered that although the definition of anaemia varies in clinical practice, the definition used in the PEGASUS trial [14] (patients with Hb levels < 10.5 g/dL) included most people who would be considered anaemic after having treatment with eculizumab or ravulizumab. The AC considered the trial results were generalisable to NHS clinical practice and demonstrated an improvement from baseline Hb levels at 16 weeks for pegcetacoplan in comparison with eculizumab.

The NICE AC agreed with the company and the ERG that differences in trial designs and key effect modifiers (Hb level and history of transfusions) could not be adjusted for using MAIC methods and that these factors could bias MAIC results. The NICE AC concluded that company MAIC results were not suitable for decision making.

The NICE AC was satisfied that the company assumption that the efficacy of ravulizumab was the same as the efficacy of eculizumab in the PEGASUS trial [14] population was reasonable.

### Cost Effectiveness

The AC concluded that the company model was suitable for decision making. The NICE AC accepted the minor revisions proposed by the ERG to use the proportions of patients undergoing chelation therapy from the PEGASUS trial CSR [28] and to include the costs of AEs within the estimates of cost effectiveness.

At the confidential prices agreed with the NHS, company base case and all company and ERG scenario analysis results showed that pegcetacoplan was more effective and less costly than eculizumab and ravulizumab.

The NICE AC noted that pegcetacoplan cost less than eculizumab and ravulizumab because pegcetacoplan is self-administered and reduces the need for blood transfusions.

### Other Factors

The NICE AC discussed the mode of treatment delivery and the frequency of treatment. The NICE AC considered that the disutility that was applied to patients treated with eculizumab in the company model (no disutility for ravulizumab or pegcetacoplan) reflects the fact that ravulizumab has the advantage of less frequent dosing than eculizumab, and that pegcetacoplan is delivered via a subcutaneous infusion which is advantageous to patients compared with intravenous infusion (eculizumab).

During the scoping phase of the appraisal, stakeholders highlighted that as pegcetacoplan is a subcutaneous infusion it can be self-administered at home which may have implications for people with physical or learning disabilities, particularly if they have manual dexterity issues. The NICE AC was satisfied that the company’s patient support programme would identify patients who need support within their home environment to be able to administer their pegcetacoplan infusions and this would help reduce inequalities of access.

### Final Guidance

The NICE AC recommended pegcetacoplan for treating PNH in adults who have anaemia after ≥ 3 months of treatment with a C5 inhibitor. The final guidance was published by NICE in March 2022 [30].

## Conclusions

Pegcetacoplan improves clinical and haematological outcomes for patients with PNH when compared with eculizumab.

Commercial access arrangements are in place for the treatment options, and therefore the incremental cost-effectiveness ratios per QALY gained are confidential; however, the NICE AC was satisfied that the most plausible ICERs per QALY gained (using the confidential discounts) for the comparison of pegcetacoplan versus eculizumab were below £20,000.

Although there was no direct evidence for the comparison of pegcetacoplan versus ravulizumab, the NICE AC considered the assumption that the clinical effectiveness of ravulizumab was the same as eculizumab was reasonable. The NICE AC was therefore satisfied that, using this assumption, the most plausible ICERs per QALY gained for the comparison of pegcetacoplan versus ravulizumab were <£20,000.

Pegcetacoplan was the first technology recommended by NICE via the low ICER FTA process.

## Declarations

### Availability of data and material

Additional data and material relevant to this appraisal are available on the NICE webpage: https://www.nice.org.uk/guidance/ta778/evidence. Data are subject to third party restrictions. In the data sharing statement for Hillmen et al. 2021 [14] (the primary study publication for the PEGASUS trial), the company state that “access to patient-level data with qualified external researchers may be available upon request and review once the trial is complete and data are published”.
